# Dr. Hull, on Intus-Susceptio

**Published:** 1802-01

**Authors:** John Hull

**Affiliations:** Manchester


GENTLEMEN,

BY giving a place in the next Number of your useful Journal to the following observations on the affections of the intestines, named *Intus-susceptio*, or *Volonlus*, you will confer an obligation on, Your's, &c.

JOHN HULL.

*Manchester, Nov.* 10, 1801.

---

IN the second volume of the Transactions of a Society for the improvement of Medical and Chirurgical Knowledge, Art. 10, Dr. Baillie has given "*An account of a singular disease in the great intestines*," which I conceive to have been a case of the Ileus *Volonlus* of Sauvages, (Nosol. Method. T. ii. p. 348, ed. 4to.) It appears to me to admit of an obvious and satisfactory explanation when considered in this point of view, and upon no other ground whatever. As it is probable that many of your Readers may not have read the Case alluded to, I am induced to present an abstract of it.

Mrs.

Mrs. R. was attended by Sir Walter Farquhar, Mr. Chilvers, and Mr. Thomas. She was unmarried, of rather a fpare habit, about fifty years of age, and had been very fubject to coftivenefs. She had been twice "attacked with vomiting, violent pain in her ftomach and bowels, and great conftipation. On both occafions, after the difeafe was removed, fhe continued for fome time extremely weak." The attack, which terminated fatally, began about the 1ft of November, 1795. "She had violent pain of the ftomach and bowels, more efpecially on the left fide, accompanied with vomiting and obftinate coftivenefs." Evacuations from the bowels were procured by powerful purgatives. Her ftools at firft were fetid, but after fome time confifted merely of blood, and were very numerous. The bloody ftools at length became mixed with an offenfive watery fluid and mucus. "About three weeks before her death, fhe voided a fubftance refembling gut, above a yard in length. For ten days before the paffing of this fubftance, and always after that time, till fhe died, fhe could have no evacuation from the bowels, unlefs held up nearly in an erect pofture." During a great part of the time, fhe could fcarcely keep any thing on her ftomach; this fymptom at length very much abated, but again became urgent towards the end of the complaint. She was feized with ftrong convulfions a few hours before her death.

The gut-like fubftance was fent to Dr. Baillie, who, on firft looking at it, fuppofed it to be a modification of coagulable lymph, thrown out by the veffels of the inner membrane of the great inteftine; but after an attentive examination, he was convinced that it was really a portion of the colon; fince in fome places the peritoneal coat was preferved, in others the appendiculæ epiploicæ and a part of the longitudinal bands were diftinctly to be feen, and he could trace the circular mufcular fibres with the moft fatisfactory degree of diftinctnefs, and could alfo obferve clearly the inner membrane. Dr. B. farther informs us, that in Dr. Hunter's collection there is a fubftance very much refembling gut, which had been difcharged during a violent purging. It is about fix inches in length, and there can be difcovered in it fome mufcular fibres, the remains of the appendiculæ epiploicæ, and a part of the longitudinal bands, which are the circumftances characterizing the ftructure of the great inteftines. The perfon from whom this portion of gut paffed away, lived two years afterwards, but the particulars of his cafe have not come to the doctor's knowledge.

From the circumftantial defcription which Dr. Baillie has given of the gut-like fubftance paffed by Mrs. R. and the engraving that accompanies it, there is the ftrongeft reafon for agreeing with this diftinguifhed pathologift, in confidering it as actually a portion of the large inteftine. The fame may be faid with refpect to the fubftance above mentioned, as preferved in Dr. Hunter's Mufæum. But his attempt to explain the manner in which the feparation of the gut in the cafe of Mrs. R. was effected, and its place fupplied, as well as fome of his other remarks upon it, do not appear fatisfactory to me.

As leave could not be obtained to infpect the body, Dr. B. acknowledges, that he cannot tell what change had taken place in the cavity of the abdomen; but he thinks it not unreafonable to fuppofe, "that a violent inflammation had taken place in a confiderable part of the great inteftine, which had thereby loft its living principle; and that, during this inflammation, a layer of coagulable lymph was formed round the gut, and enclofed it as a cafe."—"This coagulable lymph," continues he, "would foon be converted into a fort of membrane, and the dead part of the gut would be thrown off. In this way a paffage would ftill remain for the evacuation of the contents of the inteftines; but as there was no mufcular power in this newly-formed canal, the patient could have no ftool except in an erect pofture, probably by the action of the abdominal mufcles."

At firft view it may appear to fome readers, that Dr. Baillie's explanation of this fingular cafe derives fome fupport from analogy, viz. from what is obferved to take place in that affection of the bones, named *necrosis*, wherein a confiderable portion of the cylindrical bone of a limb has feparated by exfoliation, and been removed, its place being fupplied by a bony cafe, formed over it. On a more attentive confideration, however, this fuppofed analogy will not hold. In Necrofis the bony cafe is not formed by any fecretion from the veffels of the bone, that is to conftitute the fequeftra, but is produced by the veffels of the foft parts, furrounding the original bone; confequently the fequeftra is never connected with the bony cafe by a continuity of veffels, or bony fubftance. Whereas, according to Dr. Baillie's explanation, the cafe or coating, which is fuppofed to become a fubftitute for the detached portion of inteftine, is ftated to be formed by the veffels of this part of the inteftinal tube, and not by the veffels of furrounding parts; and confequently muft be ftrongly adherent to the peritoneal coat of the inteftine throughout its whole length, by means of continuous veffels, and muft alfo derive the whole or a great part of its circulating fluids from thence. Now, though it may be admitted without hefitation, that a violent inflammation may occafion the production of a thick coat of coagulating lymph upon a part of the colon, exceeding a yard in length, it appears highly improbable to me, that the feparation of fo large a portion of the great inteftine could be detached from the newlynewly-formed coat, and that this coat ſhould retain its vital principle, and become a ſubſtitute for the ſeparated portion. I know of no inſtance, wherein a membrane or viſcus, which in conſequence of inflammation had been coated with coagulating lymph, became dead, was detached from this newly-formed coat, and had its place ſupplied thereby.

On the other hand, we know that patients have ſurvived the ſeparation of very large portions of inteſtine, that had become gangrenous in conſequence of incarcerated hernia. The caſe of M. White, related by Cheſelden, in his *Anatomy of the Human Body*, furniſhes a ſtriking example. This poor woman had an umbilical hernia, and though ſhe loſt twenty-ſix inches and a half of gut, that had mortified, ſhe lived many years afterwards. Hence we might be led to conceive, *a priori*, that an extenſive portion of inteſtine, becoming intus-ſuſcepted and ſtrangulated, might be ſeparated and diſcharged *per anum* without proving immediately fatal; and if the continuity of the canal were perfectly preſerved by means of adheſions at the point where the ſeparation took place, that the patient might live a conſiderable time after an accident of this kind. But this matter does not reſt merely on conception or reaſoning *a priori*; it is confirmed by actual obſervation, as will appear from the following abridged account of a caſe, communicated by Dr. Cullen to the late Prof. Monro, and publiſhed by the latter in the ſecond volume of *Essays and Observations Physical and Literary*. This caſe occurred to Mr. Muir, a ſurgeon at Glaſgow. The ſubject of it was a boy about twelve years of age; who, after experiencing, for about a year, colic pains, diarrhœa, &c. paſſed a livid membranous ſubſtance by ſtool. Mr. Muir obſerving this to be tubular, tied one end of it, and by blowing into the other, diſtended it into a convoluted tube thirteen inches long. It had the meſentery connected to all its concave ſide, and was therefore an entire piece of inteſtine, not the villous coat only. Some ſkins of potatoes, which the boy had eaten after this piece of gut came away, were obſerved among his fæces; whence it appeared, that the continuity of the alimentary canal was not deſtroyed. The body was opened by Mr. Muir. "The folds of the inteſtines and omentum were all glued together by a fatty curdy matter. Within four inches of the valve of the colon, the ilium A, B, C, fig. 2, tab. vii. formed into the uſual curve by the meſentery D, ſuddenly roſe perpendicularly at E, where it was much contracted, and had the appearance of a cicatrice. When the inteſtine was opened, this contracted part of it was found much thicker and harder than it was any where elſe, eſpecially on one ſide, where it ſtood ſo far into the cavity as to leave a

 very very ſmall paſſage for the aliment. Along this contracted part, the meſentery F, was firm and thick. After this the inteſtine G, became of a natural enough form and make." A figure of the portion of gut, which was paſſed by ſtool, is alſo given in the ſame plate. The gentlemen in Glaſgow were of opinion, that the part of the inteſtine inflated by Mr. Muir, was an intus-ſuſcepted portion, that had been ſeparated in conſequence of gangrene, and Prof. Monro agreed with them. That a ſimilar circumſtance took place in the above caſe of Mrs. R. I have not the leaſt doubt, and I believe that, if her abdomen had been opened, the direction of the colon would have been found much altered, owing to the loſs of about half its uſual length.

From the ſmallneſs of the number of inſtances recorded, wherein a large portion of the entire ſubſtance of an inteſtine has been diſcharged by ſtool, and as ſhewing the wonderful reſources of the ſyſtem, the above three caſes are particularly intereſting.

Inteſtinal intus-ſuſceptions vary much in their extent, ſituation, and other circumſtances. It has been aſcertained by diſſection, 1ſt. That this accident may occur in one or more parts, without producing any ſymptoms, indicating obſtruction or deranged action of the inteſtines; and it is probable, that ſlight intus-ſuſceptions are frequently produced, and ſpeedily redreſſed by the periſtaltic motion of the inteſtines, where no ſuch circumſtance is ſuſpected.* 2. That intus-ſuſceptions of conſiderable extent may ſubſiſt for a conſiderable length of time, occaſioning pain of the bowels and coſtiveneſs, &c. without any adheſions taking place betwixt the receiving and received parts. 3. That where intus-ſuſceptions have continued for a great length of time, the parts have become very much confuſed and ſtrongly adhering to each other, with the paſſage through them very much contracted.† Where the intus-ſuſcepted portion of inteſtine becomes ſtrangulated or inflamed, the progreſs of the diſeaſe may be expected to be as rapid as that of ordinary caſes of enteritis, or incarcerated hernia.

As far as I know, there is no ſymptom, or concourſe of ſymptoms, which will enable us to determine the preſence of *volvulus*, much leſs the point of the inteſtinal canal in which this is ſituated, or the ſtate of the intus-ſuſcepted portion of inteſtine, except when it occurs in the inferior part of the rectum. It muſt ever be a great ſatisfaction to a practitioner, in treating

* See Haller Elem. Phyſiol. tom. vii. p. 94.

† See Eſſ. et Obſ. Phyſ. et Liter. vol. ii. p. 359, &c.

treating affections of the bowels, accompanied by obftinate conftipation, to be able to afcertain the caufe of the obftruction; but, independently of this confideration, our inability to determine the prefence of the caufe in queftion, when it occurs in the fmall inteftines, or even in the higher part of the large inteftine, is the lefs to be regretted, fince we fhould derive little if any advantage from it with refpect to the cure. If we could become acquainted with the ftate of the intus-fufcepted inteftine, it would undoubtedly affift us in our prognofis; thus it muft be obvious to every one, that the patient will have a greater chance of recovery when there is a fimple intus-fufception than where this is complicated with adhefion of the parts, or ftrangulation.

Where the intus-fufcepted portion of inteftine is a part of the large inteftine, it may defcend fo low as to be reached by the finger introduced into the anus, or it may be protruded through the anus. The latter cafe may be properly confidered as conftituting a diftinct and dangerous fpecies of exania, to which the trivial name *intus-suscepta* may be given, to diftinguifh it from the common *prolapsus ani*, wherein the lower portion of the rectum only is inverted and rendered confpicuous. A cafe of this kind occurred to me feveral years ago, in a child about twelve months old. It had been treated as a common prolapfus, and when I was called in, the inverted inteftine protruded about three inches, was livid and gangrenous. It was very eafily pufhed within the anus, but could not be properly reduced, and was conftantly forced out again by the efforts of the child. The child was too much reduced to afford me any expectation of its furviving the feparation of the mortified part, and died on the fucceeding day. An interefting cafe of this kind is related by Profeffor Monro, in his valuable paper on *Procidentiæ Ani, &c.* inferted in the 2d volume of the *Ess. & Obs. Phys. & Liter.* p. 353, &c. It was under the care of Mr. A. Drummond and himfelf. "The inverted inteftine ftood out four inches from the anus, without being much fwelled, or of a deep red colour, and the child feemed to have no other difeafe." The tumor was eafily introduced within the anus, and in this fituation they "found the orifice of the inverted gut refembling the feel of the *os tincæ* of an impregnated womb." As it could not be reduced to its natural fituation by the finger, they injected a large quantity of milk and water forcibly by a fyringe; and this operation being repeated feveral times in vain, a very long probe of whalebone was made, a fponge faftened round the probe, and moiftened with oil; the probe was introduced into the orifice, refembling an os tincæ, the fides of which refted on the fponge; with this the inteftine was pufhed a great a great way up into the body in the direction of the rectum, but without succefs. Several attempts of the fame kind failed, and the child, being attacked with fevere vomiting and perpetual tenefmus, died in a few days. Mr. Drummond, on opening the body, found that the inverfion began a little below the upper part of the figmoid flexure of the colon, and that the mefocolon was torn away from the inverted part.

It has been faid that the Baronefs de Lundi was cured of an intus-fufception by Gaftrotomy; but it can fcarcely be conceived that any practitioner would perform the operation with this view. Quickfilver, and other ponderous bodies, have been thought to be well adapted to the cure of this complaint. I am convinced, however, that little if any advantage can be derived from their exhibition. If the difeafe be feated in the large inteftines, the copious and forcible injection of air, or liquids, feems to promife the moft fuccefs. In whatever part of the inteftinal canal the intus-fufception occurs, upon fome occafions antifpafmodics and cathartics, upon others antiphlogiftics will be indicated.

The following obfervations on the cure of this complaint are fo judicious, that I am induced to conclude this paper with them: "Ilei *ab intus-susceptione* orti cura, fi quæ datur, certe eft difficillima, neque ulla alia methodus indicata videtur, quam quæ inflammationi & fpafmo convenit. Quam pro intus-fufceptione dirimenda inftitutum effe ferunt, abdominis fectio, infuperabilibus premitur difficultatibus; vix enim unquam fpecialis locus detegi, nec fine imminenti periculo vitium inteftini inflammati ad partes vicinas adglutinati indagari illoque occurri poterit. Hydrargyri hauftus ad folvendam intus-fufceptionem inteftinalem æque ambiguus videtur. Clyfmata tamen expandentia & aërea fparfim optatum effectum præftiterunt. Naturæ quoque viribus magnam canalis inteftinalis partem fufceptam ac corruptam feparari, per anum eliminari, tubique integritatem reftitui poffe, mirabilibus exemplis conftat." CALLISEN *Syft. Chir. Hod. Part. Poster.* p. 287.

---

### *To the Editors of the Medical and Phyfical Journal.*

GENTLEMEN,

